# Discrete element models for understanding the biomechanics of fossorial animals

**DOI:** 10.1002/ece3.9331

**Published:** 2022-09-16

**Authors:** Hao Gong, Joash B. Adajar, Léa Tessier, Shuai Li, Leno Guzman, Ying Chen, Long Qi

**Affiliations:** ^1^ College of Engineering, South China Agricultural University Guangzhou Guangdong Province P. R. China; ^2^ Guangdong Laboratory for Lingnan Modern Agriculture Guangzhou Guangdong Province P. R. China; ^3^ Department of Biological Science University of Manitoba Winnipeg Manitoba Canada; ^4^ Department of Biosystems Engineering University of Manitoba Winnipeg Manitoba Canada; ^5^ Department of Civil Engineering University of Manitoba Winnipeg Manitoba Canada

**Keywords:** badger, biomechanics, burrowing, discrete element method (DEM), soil

## Abstract

The morphological features of fossorial animals have continuously evolved in response to the demands of survival. However, existing methods for animal burrowing mechanics are not capable of addressing the large deformation of substrate. The discrete element method (DEM) is able to overcome this limitation. In this study, we used DEM to develop a general model to simulate the motion of an animal body part and its interaction with the substrate. The DEM also allowed us to easily change the forms of animal body parts to examine how those different forms affected the biomechanical functions. These capabilities of the DEM were presented through a case study of modeling the burrowing process of North American Badger. In the case study, the dynamics (forces, work, and soil displacements) of burrowing were predicted for different forms of badger claw and manus, using the model. Results showed that when extra digits are added to a manus, the work required for a badger to dig increases considerably, while the mass of soil dug only increases gradually. According to the proposed efficiency index (ratio of the amount of soil dug to the work required), the modern manus with 5 digits has indeed biomechanical advantage for their fossorial lifestyle, and the current claw curvature (25.3 mm in radius) is indeed optimal. The DEM is able to predict biomechanical relationships between functions and forms for any fossorial animals. Results can provide biomechanical evidences for explaining how the selective pressures for functions influence the morphological evolution in fossorial animals.

## Graphical abstract

The dynamics (forces, work, and soil displacements) of burrowing by North American Badger were predicted for different forms of badger claw and manus, using numerical models. The results indicate that the modern manus with 5 digits has indeed advantages. The findings provide biomechanical support for the evolutionary optimization of badgers’ manus for their fossorial lifestyle.

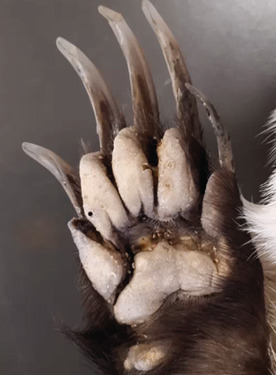

## 1 INTRODUCTION

Evolutionary morphology is often hypothesized to be adaptive to habit use. For fossorial animals, capturing prey, storing food, seeking safety, and raising young all involve underground activities (Hildebrand, 1974; Lindzey, 2003; Long, 1999). Consequently, the morphological adaptations of the body parts (e.g., head and neck, claws, manus, pedis, limb, and the entire body) would take place in the evolution of these species to sustain long‐term activities, including moving through substrates and constructing underground tunnel systems (Hamrick, 2003; Reed, 1951; Shimer, 1903). These activities involve intensive biomechanics, such as producing and transmitting forces, and resisting loads (Hildebrand, 1985). Based on the form‐function correlation paradigm (Vassallo et al., 2019), we can interpret that evolutionary morphology may have a close relationship with the biomechanical uses. Therefore, the biomechanics in relation to morphology is important for explaining why some animal body parts have evolved into their current forms, and whether further evolution would occur.

In existing animal biomechanics studies, video imaging techniques have been intensively used for capturing the motions of animals (Brainerd et al., 2010; Che & Dorgan, 2010). Another existing method is using instruments (such as tracking devices and accelerometers) to monitor animal movements (Noonan et al., 2015). The subsequent data analyses can be done using machine learning to examine different postures and movement intensities (Chakravarty et al., 2019). Computational biomechanical modeling approaches have also been used to develop musculoskeletal models to simulate the kinematics and kinetics of animal resulting from motions. A musculoskeletal model was developed to provide 3D estimates of muscle actions of extant crocodiles (Wiseman et al., 2021). Other musculoskeletal models were proposed to quantify the changing in fiber length of body tissues of a small ground‐dwelling bird for walking and running (Bishop, Michel, et al., 2021) and the vertical jumping performance of the bird (Bishop, Falisse, et al., 2021). These existing studies focused on the kinematics and kinetics of animal motions.

There have been limited studies focusing on motion‐caused forces exerted by the physical environment. Among the few studies, the energy balance principle was used to investigate contact forces between animals and surrounding substrate (e.g., sand, soil, and sediment) in the burrowing process of ghost crab (Springthorpe, 2016) and tylos granulatus (Brown & Trueman, 1996). Measurements of contact forces were conducted using custom‐built force transducers for gophers (Crisp et al., 2019), lizards (Morinaga & Bergmann, 2020), reptiles (amphisbaenians) (Navas et al., 2004), and caecilian (O'Reilly et al., 1997). In these existing studies, the complex animal systems were simplified in the analyses of motion, energy, and force; animals were either isolated from the natural environment or were induced in an artificial environment. These have hindered the advancement of animal biomechanics. Fortunately, numerical methods have advantages of dealing with complex geometries and environment. A well‐established numerical method, the finite element analysis (FEA), has been used to understand the biomechanics and evolution of animals, as reviewed by Rayfield (2007). FEA is able to analyze stress and strain in a digital structure to address questions of form‐function. For example, the stress and strain response to muscle loadings was obtained using the FEA method for different forms of reconstructed mammal jaws (Morales‐Garcia et al., 2019). FEA in vertebrate biomechanics has been also reviewed from model development to model validation (Ross, 2005). The review covered the capabilities of FEA in dealing with structure–function relationships of complex shapes, and animal growth, development, and evolution. However, FEA is suitable only for continuum material, that is, only a small deformation of the material is allowed. This limits the application of this method to animal burrowing that causes large deformations of the substrate. A newer numerical method, the discrete element method (DEM), can overcome this limitation, while offering the same capabilities. DEM is a particle‐based method, and it is suitable for discontinuum material, that is, allowing for large displacements of particles. Compared with FEA, another disadvantage of DEM simulation is the ability of providing more insights into the micromechanics of individual particles. This is particularly important when dealing with micro‐scale interaction of animal with substrate.

DEM was originally developed by Cundall and Strack (1979). A collection of discrete particles is utilized to simulate a particle assembly. The discrete particles can represent a free‐flowing material (like dry sands), or a solid material (like a rock) by bonding individual particles together. When subjected to motions and external loadings, particles interact with each other, resulting in particle displacements and forces arising at contacts between particles. A DEM model requires the user to define the properties of particles and the contact behavior between particles. Properties of particles, such as stiffness and friction coefficient, can be varied, depending on the material to be simulated. Contact behaviors between particles, such as cohesion and viscosity, can be varied using different contact models (Potyondy & Cundall, 2004), also depending on the material to be simulated. DEM formulation uses an explicit numerical scheme wherein particle interactions are detected contact by contact, and the motion and contact force of particles are calculated particle by particle. This allows obtaining the dynamic response of individual particles (micro‐level) and the assembly of particles (macro‐level). However, DEM has some drawbacks, including the requirement of a large number of particle and particle‐particle contact parameters. Various methods have been used on selections/calibrations of these parameters, although a standard method in this regard is still under development.

Due to its capabilities, DEM has been used in simulating dynamic processes involved in various fields. However, there were only few DEM‐related studies in animal biomechanics. The interaction of a bear claw with soil was previously modeled using DEM (Li et al., 2016). The model predicted the soil resistance to the claw and soil disturbance by the claw. The purpose of that study was to investigate whether the bear claw's shape could be adapted into plow design in agriculture. In a similar application, a DEM model was used to predict the soil resistance to a five‐claw combination of a mole (Yang et al., 2018). The model simulated soil cutting by the claws at different soil cutting angles (rake angles) travel speeds. Although these studies have proved that the DEM is a promising tool for modeling the interactions of animal claws with their physical environment, the abilities of DEM need to be tested in the context of animal burrowing, where manus is engaging with substrate in different motion patterns. The soil particle size (8 mm in diameter) used in Yang et al. (2018) was large relative to the size of the claw, which may affect the accuracy of the model results. Finally, the existing soil‐claw models were lacking of validations. This study aimed to fill all these gaps.

**FIGURE 1:**
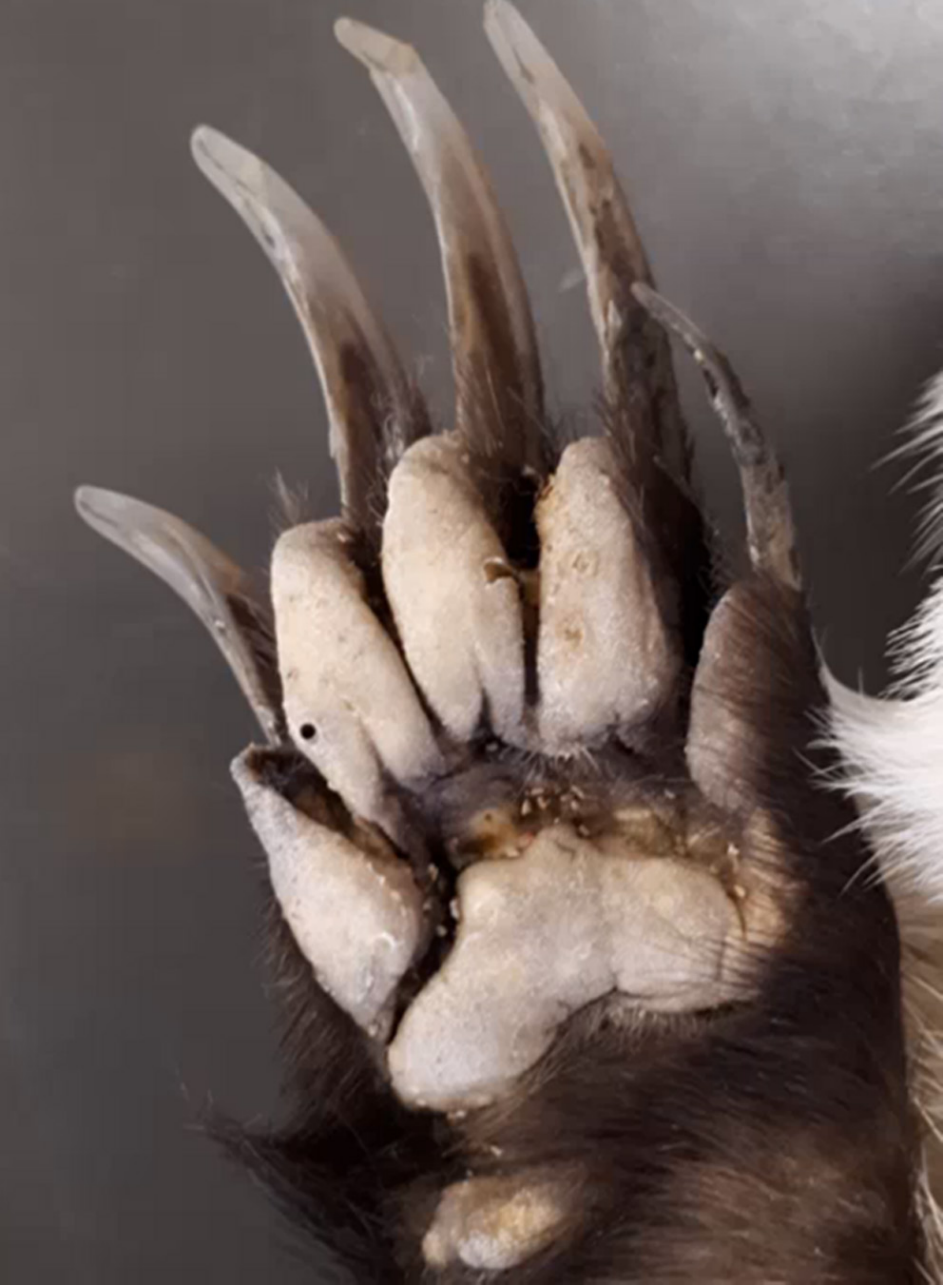
A badger manus specimen provided by Beaty Biodiversity Museum (University of British Colombia, Vancouver, Canada).

We started with proposing a general biomechanical model using the DEM to simulate the interaction of an object (representing any animal body part) with a substrate. Then we applied the general model in the case of burrowing by the North American badger (*Taxidea taxus*), a highly active animal in underground movement (Noonan et al., 2015). The main body parts of badger for burrowing are the manus and claw (Figure 1), which must have experienced a long history of adaptation and evolution in the morphological features in improving the burrowing performance (Hopkins & Davis, 2009; Stein, 2000). Thus, we focused on modeling manus‐soil and claw‐soil interactions using the DEM, and simulating artificially reconstructed forms of manus and claws. These allowed us to assess the abilities of the DEM in dealing with complex geometries and motions as well as the changes in morphology. The results provide biomechanical insights into the evolutionary adaption of fossorial animals in response to selection for improved survival abilities.

## 2 MATERIAL AND METHODS

### 2.1 Development and validation of an interaction model

**FIGURE 2:**
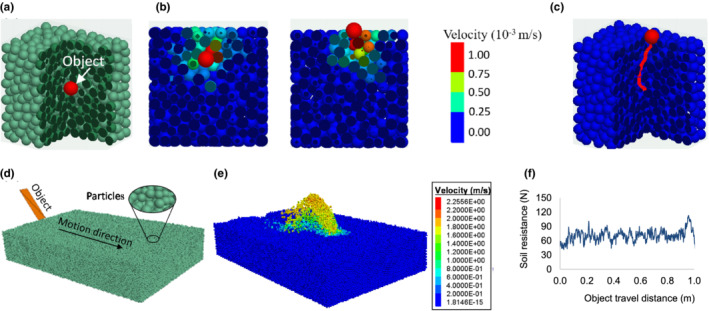
Interaction model. (a) Substrate domain and a simplified animal body part (a spherical object) submerged in a substrate. (b) Velocity contour of substrate particles resulting from the object's motion. (c) Trajectory of the object in the substrate. (d) Substrate domain and the initial position of a simplified animal body part (a rectangular object). (e) Velocity contour of substrate resulting from the object's motion, showing the disturbance of substrate particles. (f) Soil resistance to the rectangular object over the distance of travel.

A general interaction model was developed using the DEM to simulate the interaction of an animal body part with the physical environment. The model was created in a commercially available DEM software package, Particle Flow Codes in Three Dimension (PFC^3D^) (Itasca Consulting Group, Inc.). In the interaction model, an animal body part is simplified as a moving object and the physical environment is simplified as a substrate. The moving object can have any geometry, moving in any motion pattern, depending on the type of animal and the nature of the animal's activity. We used an animal body part of a simple spherical shape submerged in a substrate domain for demonstration (Figure 2a). The substrate domain was an assembly of discrete particles. Animal body can be given an any motion pattern that reflects the real motion trajectory of the animal. For simplicity, the spherical object was specified a vertical velocity in the z direction, and a zero velocity in the x and y directions, meaning that the object was let move upward freely to mimic the motion of an animal. As the spherical object moves through the domain, it impacted the surrounding substrate particles (Figure 2b). The resultant displacements and velocities of the particles can be monitored. The contact forces between animal and substrate particles can also be monitored. The sum of the contact forces between the animal and substrate particles in the motion direction of the animal is named as substrate resistance. Substrate resistance is of interest, as the force required for a fossorial animal to overcome the substrate resistance is biologically limited by its muscle architecture and function capacity (Moore et al., 2013). Furthermore, the motion trajectory (Figure 2c) of the animal can be traced over time.

DEM is capable of simulating complex geometries, for examples, various types of soil‐engaging tools (Zeng et al., 2020) and sophistically shaped hoe openers (Murray & Chen, 2019) in earth‐moving applications. In these studies, simulation results have been validated using measurements. The capability of the DEM in simulating various geometries in various motion patterns is further demonstrated here by the burrowing process of animals. In the burrowing process, animals use different digging apparatuses, such as teeth, head, and neck (Lacey et al., 2000). These apparatuses interact with different substrates. To make the model general, a digging apparatus of a rectangular shape was used for simplicity. Again, for simplicity, the motion of the digging apparatus was fixed to be a linear motion in the horizontal direction (Figure 2d). The moving rectangular object resulted in substrate particles flowing upwards and laterally around the object (Figure 2e). The amount of disturbed substrate particles reflects the amount of work done by animal. Thus, it is an interesting indicator of burrowing performance, and it can be predicted by the model, as described later in the soil‐claw and soil‐manus models. This burrowing application was used to validate the interaction model. In the validation, we used soil as the substrate, and soil particles were assumed to be spherical. The model was run to predict the soil resistance to the rectangular object (Figure 2f). The predicted soil resistance forces were comparable with the soil resistance forces measured in the experiment. The details of the experiment and validation process are described in detail in the Appendix S1.

**FIGURE 3:**
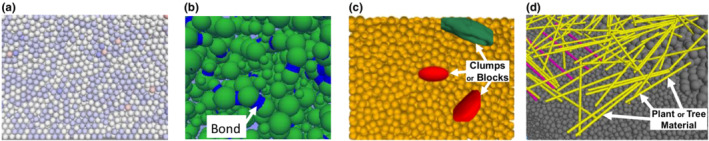
Examples of modeling various conditions of substrate. (a) Sand particles with a uniform diameter. (b) Cohesive soil with bonds between particles. (c) A stony soil. (d) Plant and tree materials on ground surface.

In addition, various physical environments (desert, forest, mountain, grassland, and farm field) where animal activities occur can be simulated by the interaction model. For example, the model domain can be an assembly of free‐flowing particles with uniform particle size to represent a dry soil (Figure 3a), or bonded particles (particles which are in contact are bonded together) to represent a cohesive soil with various clod sizes (Figure 3b). In the DEM, unbreakable clumps or blocks of any shape can be integrated into the domain to represent a stony soil condition (Figure 3c), or using breakable clusters represents plant or tree materials on ground surface (Figure 3d). In terms of domain size, it can be large or small, allowing us to custom set the space of interest where animal activities occur. Substrate properties affect energy requirement of burrowing (Vleck, 1979) and aforementioned dynamic attributes. Physical properties (e.g., particle size, particle density, and bulk density) and mechanical properties (e.g., stiffness and friction coefficients) of substrate can be selected or/and calibrated to reflect the real‐life conditions to be simulated. The particle‐particle contact model can be defined to reflect the behavior of the substrate material to be simulated (Potyondy & Cundall, 2004).

### 2.2 Application of the interaction model to burrowing

The interaction model was applied to simulate the burrowing process by the North American badger in soil. The observation on live badgers by Quaife (1978) showed that the burrowing process involved two stages: soil cutting and digging. Soil cutting is performed mainly by the claws that mechanically penetrate and loosen a hard soil surface, while soil digging involves the entire manus that transfers and removes loose soil. Thus, the interaction model was extended to simulate these two stages. The model parameters for soil are those listed in Table S1. Claw‐soil contact parameters were regarded as soil–soil contact parameters. This assumption was made based on the principle of “weaker” material (soil in this case) governing the behavior of the interaction in DEM models (Gong et al., 2022).

**FIGURE 4:**
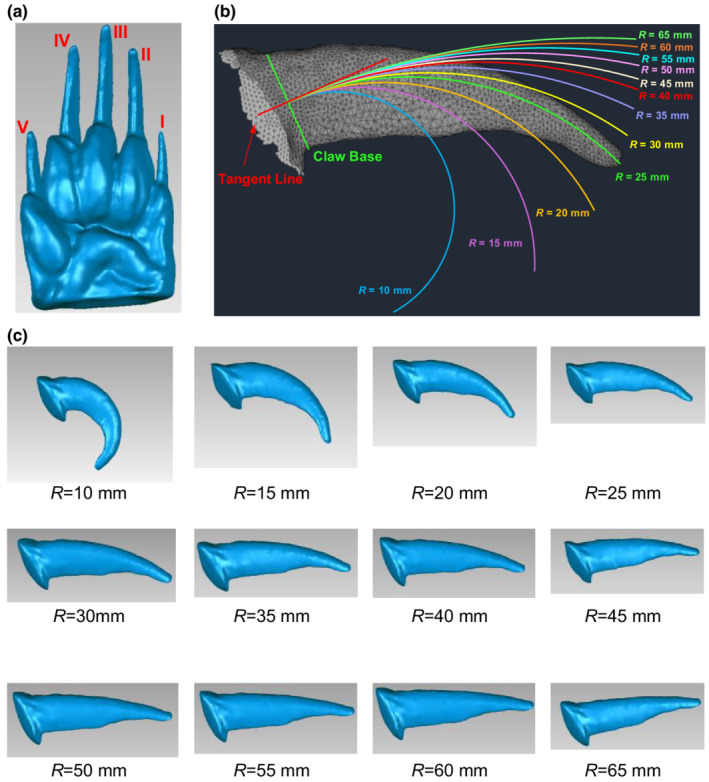
Badger manus and claw. (a) Example of a manus from a badger specimen and a 3D scan model. (b) Curvatures generated from the representative specimen of claw (Specimen No. 12, digit III). (c) Artificial claw models used for simulations.

Fifteen North American Badger specimens were provided by the Beaty Biodiversity Museum (University of British Colombia) (Table S2). A manus as shown in Figure 1 from each badger specimen was scanned using an XTOM‐MATRIX Blue Light 3D Scanner (XTOP 3D Technology Co., Ltd.). After 3D scanning, the image files were imported into the software, *Geomagic Studio*, to obtain the 3D models of manus. A 3D scan model is shown in Figure 4a, and the rest, together with the actual manus, are shown in (Figure S3). In addition, morphological characteristics of the badger manus and claw were obtained. Each of the five digits on the manus was given a number (Figure 4a). The results of manus width and thickness, the claw length, width, and thickness are discussed in Appendix S1.

#### 2.2.1 Simulation of soil cutting by a claw

Soil cutting was simulated using the interaction model in which the object is a badger claw and the substrate is soil. To examine the effects of claw morphological characteristics (form) on soil cutting performance (function), artificial claws with different radii of curvature (defined in Figure S4d) were created based on features of the most representative claw (Specimen No. 12) (Figure 4b). The dimensions of this specimen had the least overall relative error (4.5%) when compared to the average values of the morphological characteristics of all 15 specimens. Then, based on this specimen, 12 different radii of curvature (*R*) values are generated, and they were 10, 15, 20, 25, 30, 35, 40, 45, 50, 55, 60, and 65 mm (Figure 4c), while keeping the arc length the same as the representative claw. This ensured that *R* was the only varying factor examined. The steps for obtaining these radii are described in Appendix S1.

**FIGURE 5:**
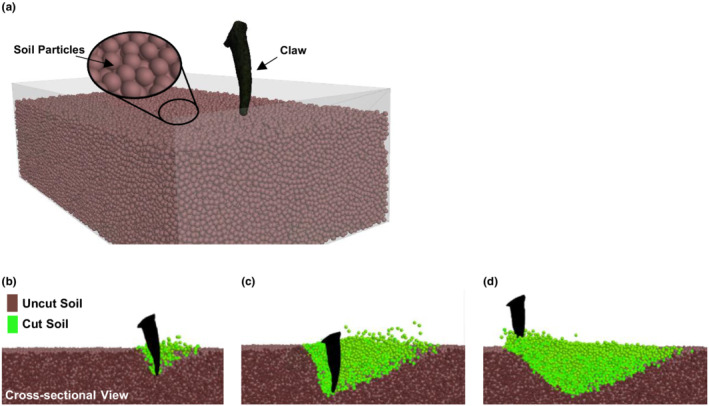
Claw‐soil interaction model. (a) Soil domain and a claw before initiating soil cutting (*t* = 0 s); (b–d) The start of soil cutting (*t* = 0.03 s), the maximum depth of cutting (*t* = 0.06 s), and completion of cutting (*t* = 0.14 s), respectively

In the claw‐soil interaction model (Figure 5a), the size of the soil domain was 200 × 100 × 50 mm to accommodate the soil cutting action of claw. Soil particles were spherical and the diameter was 2 mm. The soil bulk density was set to be 1900 kg/m^3^, a typical value for hard soil at the ground surface (Das, 2009; Quaife, 1978). The claw was set with the real‐life cutting motion patterns (Figure S7c–e). As described in the Appendix S1, the motion pattern was developed based on the data of the digging motions of a live North American badger, recorded by Quaife (1978). The soil surface elevation was set at 177 mm below shoulder level to ensure that the claws could be embedded up to their proximal end. As the claw penetrates the soil, soil was cut (Figure 5b). More soil was cut as the claw moves (Figure 5c,d). The total mass of cut soil was determined after the claw is lifted from the soil (Figure 5d). It was calculated by multiplying the total volume of displaced particles by the soil particle density. The resistance force from soil to the claw was monitored over time, as the claw moved through the soil. The resistance force was the sum of the contact forces between the claw and soil particles in the motion direction of the claw.

#### 2.2.2 Simulation of soil digging by a manus

**FIGURE 6:**
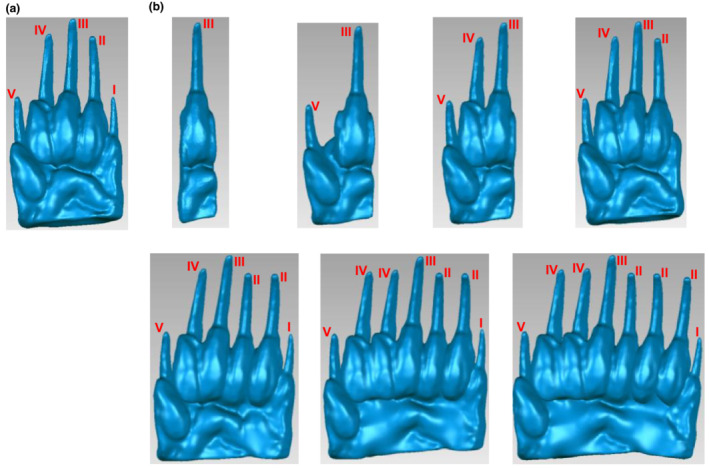
Badger manus models. (a) The 5‐digit manus from the 3D scan model of specimen No. 12. (b) 1‐, 2‐, 3‐, 4‐, 6‐, 7‐, and 8‐digit artificial manus in succession

Soil digging was simulated using the interaction model where the object was a badger manus and the substrate is soil. To examine the effects of manus morphological characteristics (form) on the digging performance (function), artificial manus with different numbers of digits were created based on the most representative 3D manus model (Figure 6a). Seven artificial manus with 1‐, 2‐, 3‐, 4‐, 6‐, 7‐, and 8‐digit (Figure 6b) were created for simulations. The considerations in creating these artificial manus are described in the Appendix S1. In the simulations, digits were made to be adducted (Figure S6a) and flexed (Figure S6b) to reflect the digging posture of badger.

**FIGURE 7:**
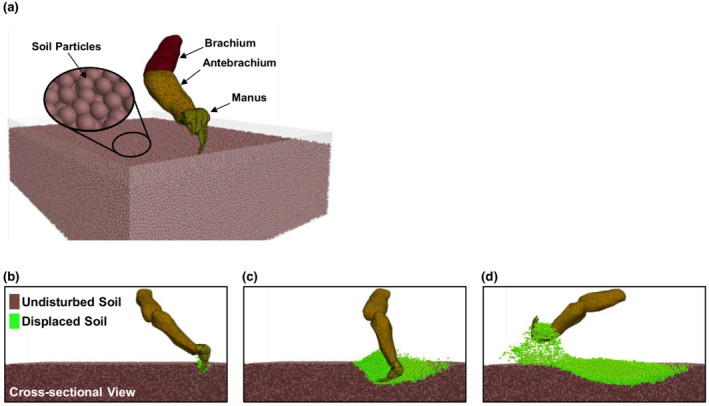
Manus‐soil interaction model. (a) Soil domain, forelimb, and manus model before initiating soil digging (*t* = 0 s). (b–d) Start of soil digging (*t* = 0.18 s), maximum depth of digging (*t* = 0.28 s), completion of digging (*t* = 0.51 s) in a power stroke.

In the manus‐soil interaction model (Figure 7a), the soil domain was 500 × 400 × 120 mm. The soil particle diameter was 4 mm, and the soil bulk density was set to be 1600 kg/m^3^ for a typical soil condition where soil digging is commonly performed (Campbel, 1985; Das, 2009; Quaife, 1978). To facilitate the soil digging, a forelimb model was created based on the morphological data of the North American badgers reported by Quaife (1978) and Moore et al. (2013). The “scooping” motion of the forelimb (Figure S7f–h) was set to be the real‐life motion. As described in the Appendix S1, the motion pattern was derived based on the data recorded for live North American badgers by Quaife (1978). The literature data also showed that the forelimb digging motion involved a power stroke (engaging with soil) and a retracting stroke (retracting the manus to the initial position). Only the motion of the power stroke was simulated in this study because no soil is displaced during the retracting stroke. The soil surface was set 180 mm below shoulder level to ensure that the majority of the soil interactions involved the manus (i.e., preventing the arms from disturbing the soil). In the simulation, as the forelimb follows through with a power stroke, the manus engages with the soil (Figure 7b), and more soil particles are being displaced (Figure 7c,d). The total mass of cut soil was determined after the manus is lifted from the soil (Figure 7d) and the resistance force of the manus was monitored, in the ways described in the claw‐soil interaction model. In addition, the equivalent work performed for a stroke was determined by measuring the area beneath the force‐displacement curve. The average number of strokes was four before the badger engaged the other forelimb or moved to a new location (Quaife, 1978). Thus, simulations were performed for each of the four consecutive power strokes of digging.

## 3 RESULTS

### 3.1 Effects of the curvature of claw on soil cutting

**FIGURE 8:**
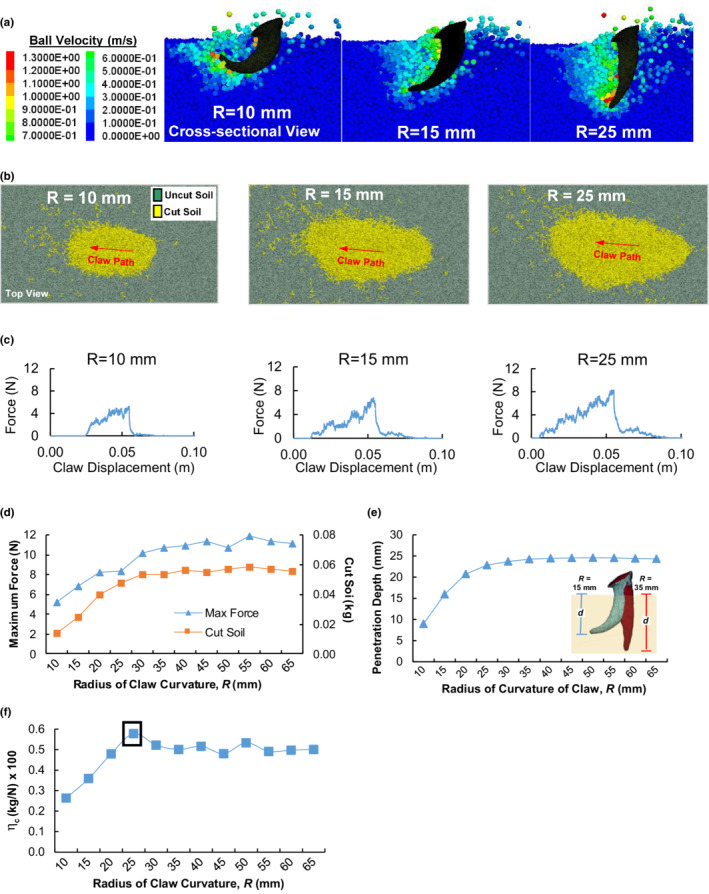
Results from the claw‐soil interaction model for different radii of curvature (*R*). (a) Cross‐sectional view of soil particle velocity contours resulting from the moving claw. (b) Top view of the amount of loosened soil at the completion of soil cutting. (c) Resistance forces experienced by claws in a soil cutting cycle. (d) Total mass of cut soil and maximum resistance force. (e) Relationship between *R* and the soil cutting depth (*d*). (f) Soil cutting efficiency, *η*_*c*_ (the ratio of the total mass of cut soil to the maximum resistance force).

Simulation results of claw‐soil interaction are presented in Figure 8a–c for three *R* values and in Figures S8 and S9 for the others. Less curved claws impact more soil particles, shown by the soil particle velocity contours during soil cutting (Figure 8a). This is also shown by a larger soil area loosened by a less curved claw in Figure 8b, the top views of the surface when the soil cutting is completed. The force‐displacement relationships show that the resistance experienced by a claw begins to increase while the claw penetrates the soil, and this force continuously increases to a peak until the maximum cutting depth is reached (Figure 8c). Subsequently, the force drops abruptly as the claw exits the soil. This force‐displacement relationship holds true regardless of the radius of curvature of the claw. However, the maximum force (the peak force) was different. Results reveal that the maximum resistance force and the mass of cut soil rapidly increase at lower *R* values and then slow down when *R* is >30 mm (Figure 8d). To explain these, we need to understand how the *R* affects the maximum soil cutting depths (*d*), as *d* significantly affects the soil dynamic attributes (McKyes, 1985). Simulation showed that the *d* varies from 9.0 to 24.3 mm over the range of *R* (Figure 8e). A less curved claw (*R* > 30 mm) reaches a much greater soil depth, and beyond *R* = 30 mm, the cutting depth is fairly constant. The mass of cut soil and the maximum resistance force experienced by claws are correlated to the trend of cutting depth.

As shown in Figure 8d, less curved claws are effective at cutting soil in terms of the mass of cut soil, but they require higher amounts of force. As such, it is essential to determine how much more force is required by the badger to cut an additional amount of soil. Therefore, the biomechanical performance of the claw was evaluated by considering both the amount of cut soil and the corresponding amount of resistance force that the claw needs to overcome. Here, we introduced the soil cutting efficiency index (*η*_*c*_). The *η*_*c*_ was defined as the ratio of the cut soil mass to the corresponding maximum cutting force. The *η*_*c*_ increases as *R* increases above *R* = 10 mm until the index reaches a peak at *R* = 25 mm (Figure 8f). The *η*_*c*_ then decreases and becomes approximately constant as *R* increases further. This reveals that when the claw has a radius of curvature of 25 mm, the least amount of force is required to cut a particular amount of soil.

### 3.2 Effects of the number of digits on soil digging

**FIGURE 9:**
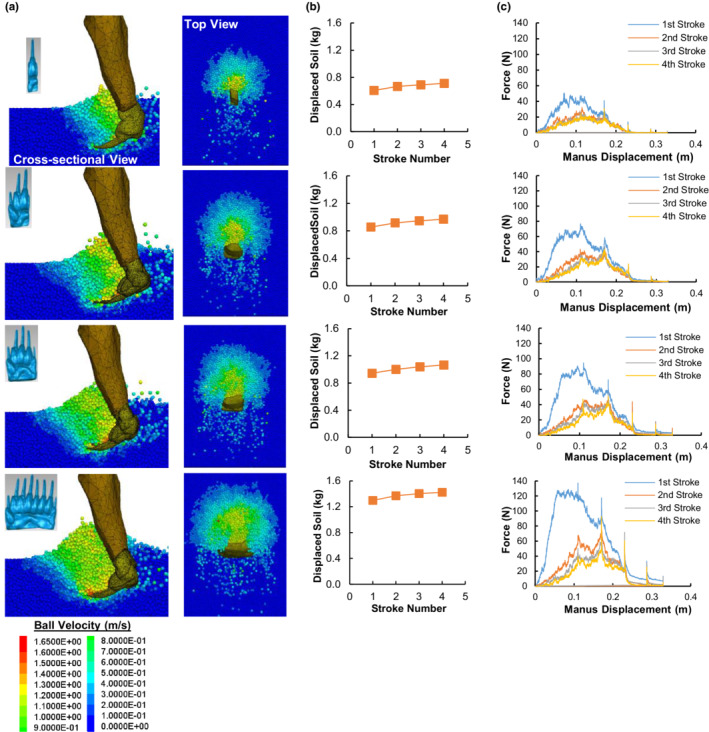
Results from the manus‐soil interaction model for 1‐, 3‐, 5‐, and 8‐digit manus from the top to the bottom in succession. (a) Soil particle velocity contours from cross‐sectional and top views. (b) Accumulated mass of the displaced soil after each of four power strokes of digging. (c) Resistance force exerted on the manus during the four strokes of digging.

Simulation results of manus‐soil interaction are presented in Figure 9a–c for 1‐, 3‐, 5‐, and 8‐digit manus, and in Figure S10 for the others. An increase in the number of digits increases the amount of displaced soil, shown by the soil particle velocity contours in both the top and cross‐sectional views of the soil during digging (Figure 9a). A large number of soil particles are moved behind the forelimb, with some particles being pushed to either side of the manus. Most of the soil displacement occurs during the first stroke, and the quantity of displaced soil accumulates with the subsequent three strokes (Figure 9b). The soil resistance force that the manus encounters increases rapidly as the manus moves through the soil (Figure 9c). Notably, a manus with more digits resulted in larger resistance forces during all power strokes. Regardless of the number of digits, the highest force is encountered during the first stroke, and a lower force is required for digging during the successive three strokes. This is because the first stroke must break apart the firm and undisturbed soil which provides more resistance against a moving object.

**FIGURE 10:**
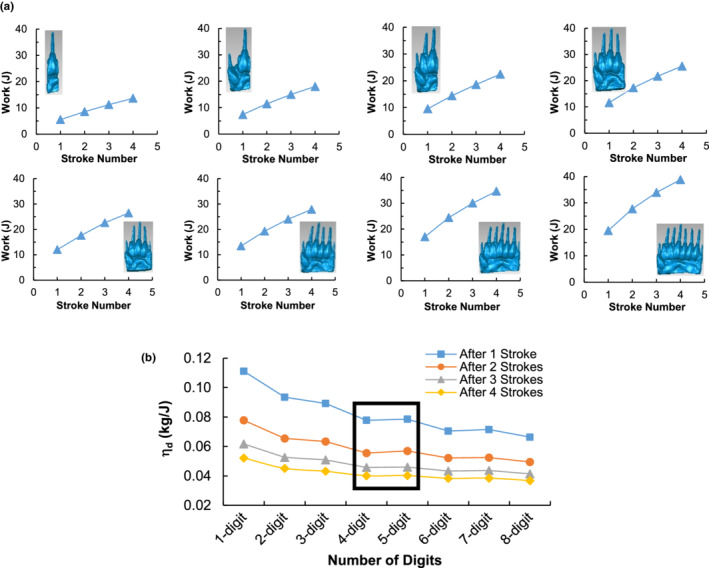
Work and digging efficiency. (a) Cumulative amount of work performed after every power stroke during soil digging by 1‐, 2‐, 3‐, 4‐, 5‐, 6‐, 7‐, and 8‐digit manus in succession. (b) Soil digging efficiency, *η*_*d*_ (the ratio of the total mass of displaced soil to the total work required for digging).

Besides the force, the amount of work that a badger needs to do in digging is also an indicator of biomechanical performance. The accumulated amount of work after every stroke of digging increases with the number of digits (Figure 10a), which ultimately suggests that a badger must employ additional work when digging with a manus having more digits. This finding suggests that a manus with more digits is a disadvantage when solely considering the work requirement. However, we found that more soil was being displaced by the manus with more digits. This concurs with the observation that most fossorial animals have a stouter and broader manus, which is beneficial for digging (Carrizo et al., 2014; Rose et al., 2014; Shimer, 1903). This finding suggests that a manus with more digits is an advantage when solely considering the mass of displaced soil.

Thus, there is a trade‐off between the amount of work required and the amount of soil displaced. Considering both, a soil digging efficiency index (*η*_*d*_) was defined as the ratio of the total mass of displaced soil to the total work required for digging. Results revealed a general decrease in *η*_*d*_ as the digit number increases (Figure 10b). In comparing the efficiency of the first stroke, a decrease (in *η*_*d*_) of as high as 60% was observed between the 1‐ and 8‐digit manus. Another observation is that the rate of decrease in *η*_*d*_ decreases as the digits increase. The most interesting finding is that the 4‐digit manus and the 5‐digit manus resulted in similar digging efficiencies. However, when compared to the 4‐digit manus, the 5‐digit manus disturbed 4.5% more soil (Figure 9b and Figure S10b). This indicates that the 5‐digit manus dug more soil without compromising efficiency.

## 4 DISCUSSION

Despite the importance of biomechanics for explaining animal behavior and morphological evolution, the related information has been limited. The main reason was lacking of the method that is able to address the large displacement of the substrate with which the animals interact. Quantifying of such a system through measurements in a real‐life setting, if possible, is difficult. The use of the discrete element method (DEM) has made it possible or easier. This has been demonstrated previously in modeling claw‐soil interactions in agricultural applications to explore whether shapes of animal claws could be adapted into the design of soil‐engaging tools for reduced soil resistance forces (Li et al., 2016; Yang et al., 2018). This is further demonstrated in modeling the burrowing process using North American badger as a case study. The interaction model developed using the DEM allowed easily integrating 3D scanned models of the real‐life manus and claw in the model. By using the real‐life motion patterns of badger in the model, the information generated through modeling was considered to represent closely the real‐life behavior of badger.

Evolutional morphology is often hypothesized to be adaptive to habit use. This hypothesis was tested biomechanically using the DEM, through comparing various artificial forms of animal body parts under the same conditions. This makes it possible to assess if a change in morphology can yield a more or less efficient or effective performance. In the case study of badger, we found that less curved claws cut through more soil and experienced higher soil resistance than more curved claws (Figure 8a–c). The proposed efficiency index indicates that the optimal curvature is 25 mm (Figure 8f). This coincides with the current claw form of badgers which had an average radius of curvature measured (25.3 mm) from 15 badger specimens. In the case study, we also found that fewer digits on a manus may be biomechanically advantageous for digging based on the work required to do (Figure 10a). This finding raises the question of why badgers have not evolved to have fewer digits. This can be addressed by the result that fewer digits dig less soil, which would require the badger to perform more strokes to dig a particular amount of soil. It was shown to disturb an average of 10% less soil for every digit lost in a stroke (Figure 9b). Hence, at any instant during digging, a manus with fewer digits will require more and faster strokes to dig the same amount of soil within the same amount of time. This is not only inefficient, but also will encounter another constraint in the moving speed of the forelimbs (Hildebrand, 1974; Polly, 2007). With each increment in the digit number, the work required to dig increases (Figure 10a) and the digging efficiency decreases (Figure 10b), yet with each decrement in the digit number, the mass of displaced soil decreases (Figure 9b). Thus, there is a trade‐off between the amount of displaced soil and the work requirement.

The DEM can enhance our understanding of the relationship between the forms and the functions, which enables us to predict the adaptive evolution of fossorial mammals. Based on our findings from the case study of a badger, we can consider the current claw and manus of the North American badger to have biomechanically advantage for its fossorial lifestyle and that any further changes in its morphology may be maladaptive, assuming unchanging biotic and abiotic environmental factors. A manus would be unlikely to gain more digits only to maximize the amount of soil dug; more digits would not only be inefficient but also require the development of a forelimb to support the increased force requirement, and this modification may compromise the animal moving speed. The general speed of a badger is still important when moving aboveground and avoiding predators (Quinn, 2008; Shimer, 1903). Although digit gain is uncommon, the reduction of digits is not (Coates, 2005). The 4‐digit manus and the 5‐digit manus resulted in similar digging efficiencies (Figure 10b), although the 4‐digit manus dug less soil. If future selective pressures deem it necessary and if there are no other significant negative impacts on digging, the loss of a digit may be possible and badgers may eventually evolve to have 4‐digit manus.

The diversity and flexibility are among many advantages of the DEM. Fossorial animals are morphologically diverse and variable in sizes and shapes. Thus, the DEM would be better suitable for modeling numerous problems traditionally dealt in the literature. In a previous study, it was observed that polychaete *Cirriformia moorei* (marine infauna) created burrows using its hydrostatic skeleton to fracture the sediment, and the stress distribution in the sediment varied with the body size and shape of polychaete (Che & Dorgan, 2010). This could be investigated more effectively by a polychaete‐sediment interaction model using the DEM. In another previous study, amphisbaenid (*Leposternon microcephalum*) compresses soil when burrowing, and the resistance force the amphisbaenid experienced varied with the body cross‐section area (Navas et al., 2004). The resistances for various body cross‐section areas could be predicted by an amphisbaenid‐soil interaction model using the DEM. Similarly, to investigate which form, snake‐like or lizard‐like body shape, was more favorable to penetrate sands, the penetration resistance forces could be predicted using a DEM model, rather than measuring the resistance forces in an artificial environment done by Morinaga and Bergmann (2020). Other examples where the DEM could be used are evaluations of excavation performance of other animals, such as ghost crabs (*Ocypode quadrata*) (Springthorpe, 2016) and pocket gophers (Crisp et al., 2019). In summary, much more scenarios of forms (e.g., body cross‐sectional areas, snake‐like or lizard‐like) and substrate (e.g., soil, sediment, or sand) could be examined by modeling animal‐substrate interactions using the DEM. Consequently, more information could be generated on the effects of morphological forms on the performance of functions (e.g., fracturing, compressing, or excavating).

In conclusion, the DEM has shown great promise to be an effective method for studying the biomechanics of fossorial animals. This has been tested in the case study of North American badgers. The general interaction model developed can be extended to any fossorial animals as the variations on morphology can be easily integrated into the model, no matter how complex their geometries are. The modeling using the DEM will predict the resultant performance of their specific functions in terms of multiple dynamic attributes. Altogether, the interaction model generates findings that argue biomechanically in favor of the form‐function paradigm. In modeling badger burrowing, soil particles were assumed to be spherical and uniform in size, and were validated against only one type of soil (sandy loam soil) and soil resistance data. These limitations need to be addressed and various types of substrates need to be tested in future studies.

## AUTHOR CONTRIBUTIONS

**Hao Gong:** Investigation (equal); methodology (equal); visualization (equal); writing – review and editing (equal). **Joash Adajar:** Data curation (equal); formal analysis (equal); writing – original draft (equal). **Lea Tessier:** Writing – review and editing (equal). **Shuai Li:** Data curation (equal); software (equal); visualization (equal). **Leno Guzman:** Visualization (equal); writing – original draft (equal). **Ying Chen:** Conceptualization (equal); investigation (equal); methodology (equal); project administration (equal); resources (equal); validation (equal); writing – original draft (equal); writing – review and editing (equal). **Long Qi:** Conceptualization (lead); funding acquisition (lead); resources (supporting).

## CONFLICT OF INTEREST

The authors declare no conflict of interest.

## Supporting information


Appendix S1

## Data Availability

Primary data are stored on Dryad (https://doi.org/10.5061/dryad.j3tx95xjc).
