# Corrigendum

**DOI:** 10.1002/ece3.9394

**Published:** 2022-10-03

In the recent article by Gong et al. (2022), the second and fifth affiliations were interchanged. The correct order of affiliations is shown below:

Hao Gong^1^, Joash Bryan Adajar^2^, Léa Tessier^3^, Shuai Li^1^, Leno Guzman^4^, Ying Chen^4^, Long Qi^1,5,*^

^1^College of Engineering, South China Agricultural University, Guangzhou, China

^2^Department of Civil Engineering, University of Manitoba, Winnipeg, Manitoba, Canada

^3^Department of Biological Science, University of Manitoba, Winnipeg, Manitoba, Canada

^4^Department of Biosystems Engineering, University of Manitoba, Winnipeg, Manitoba, Canada

^5^Guangdong Laboratory for Lingnan Modern Agriculture, Guangzhou, China

The authors apologize for this error.
