# Supplementary material for: Discrete element models for understanding the biomechanics of fossorial animals
Source: Ecol Evol. 2022 Sep 16;12(9):e9331. doi: 10.1002/ece3.9331 (PMC9481867; doi:10.1002/ece3.9331)
Supplement: Supplementary file 1 — Appendix S1 [file ECE3-12-e9331-s001.docx]

# SUPPORTING INFORMATION

## Soil bin experiment

An experiment was conducted to measure soil resistance to an object for validation of the general object-soil interaction model. In the experiment, the object selected was a flat rectangular object (5 mm thick, 50 mm wide, and 200 mm long). The object was tested in an indoor soil bin facility at the University of Manitoba, Winnipeg, Canada. The soil bin was 1.0 m wide, 0.6 m deep, and 10 m long (Figure S1a). The soil was a sandy loam (70% sand, 16% silt, 14% clay). This is the soil type commonly found in the habitats of badgers (Apps et al., 2002; Quinn, 2008). The rectangular object was mounted onto a shank at an inclined angle of 50° (Figure S1b). The shank was connected with the carriage of the soil bin through a dynamometer. The dynamometer was designed by Mahadi et al. (2017) to measure the soil resistance force when an object passes through soil.

The soil was prepared before each test to create uniform soil conditions for all the tests. Firstly, the soil was tilled once using a sweep cultivator, then was levelled using a blade leveller, and finally was compacted with two passes of a roller. The soil moisture content was 21.1% (d.b.), and the soil bulk density was 1427 kg/m^3^. To test, the object was submerged into the soil at the desired depth, and the dynamometer was turned on in order to start recording data. Then the soil bin carriage was given a travel speed of 5 km/h. This speed was close to the average linear motion speed of badger manus, estimated based on the information provided by Quiafe (1978). Quiafe provided rotational velocities information and we used them to estimate the corresponding translational velocities of the different body parts. The estimated translational velocities varied from 3.34 to 6.05 km/hr with an average of 5.2 km/h. Once the object had travelled to the end of the soil bin, the dynamometer was turned off and the force data were retrieved. Tests were conducted for each of the submerging depths of 75 and 125 mm. These two depths were selected arbitrarily. Different animals dig to different depths. Even within an animal, digging depth will change over the time of digging, likely varying from shallow to deeper. Further research is required on a wider range of depths.

| **(a)** | **(b)** |
| --- | --- |
|  |  |

**FIGURE S1** Testing of the rectangular object in the soil bin testing facility. (a) Back view of the object and dynamometer for measuring the soil resistance to the object (b) Side view of the object and the set inclination angle of the object

## Validation of the interaction model

The interaction model was validated using the soil resistance data from the experiment. Soil resistance forces were predicted using the object-soil model shown in Figure 1d. The model soil domain was 800 mm long, 500 mm wide, and 50 mm deep. The soil particle diameter was 4 mm. The total number of soil particles in the domain was 1,016,521. The bulk density of the soil was set the same as that in the experiment. The soil particle parameters (Table S1) were adopted from (Sadek & Chen, 2015) who studied the same soil. The rectangle object had the same geometry, inclination angle, submerging depths, and travel speed as in the experiment. Through running the model, the soil resistance forces were predicted. The predicted soil resistance forces were compared with the experimental data. Before comparisons, the resistance forces were averaged over the travel distance for each test or simulation run. Then the average resistance forces were compared (Figure S2). Both the experiment and prediction showed that the rectangle encountered a higher resistance at a greater submerging depth. This observation is consistent with the soil dynamic theory of earth moving (McKyes, 1985). At the 75 mm cutting depth, the predicted average force was slightly higher than the measured one. At the 125 mm, the predicted and measured forces were well matching. Overall, the predicted and measured soil resistance forces had a good agreement with a relative error of 7.8% over the two soil cutting depths.

**TABLE S1** Medium-textured soil parameters used for the simulations (Sadek & Chen, 2015)

| Parameter | Unit | Value |
| --- | --- | --- |
| Particle Modulus of Elasticity (*E_p_)* | Pa | 2.5e5 |
| Friction Coefficient (*µ*) | - | 0.5 |
| Bond Modulus of Elasticity (*E_b_*) | Pa | 2.5e7 |
| Bond Normal Strength ($\bar{\sigma}_{c}$) | Pa | 2e4 |
| Bond Shear Strength ($\bar{c}$) | Pa | 2e4 |
| Bond Radius Multiplier | - | 0.5 |
| Bond Gap | - | 0 |
| Local Damping Coefficient (*α*) | - | 0.5 |
| Viscous Damping Coefficient (*β*) | - | 1.0 |

**FIGURE S2** Comparisons of the average soil resistances between the model predictions and measurements

## Badger specimens and 3D scans of the manus

Fifteen North American Badger specimens were provided by the Beaty Biodiversity Museum (University of British Colombia, Vancouver, Canada), and they are described in Table S2. Manus specimens and their 3D models are shown in Figures 3a and S3.

**TABLE S2** Descriptions of the badger specimens provided by the Beaty Biodiversity Museum (University of British Colombia, Vancouver, Canada)

| Specimen No. | Scanned Manus | Sex | Location | Year | Specimen condition |
| --- | --- | --- | --- | --- | --- |
| 1 | Right | Male | BC | 1952 | Pelt with manus attached |
| 2 | Right | Female | BC | 1993 | Pelt with manus attached |
| 3 | Right | Male | AB | 1945 | Pelt with manus attached |
| 4 | Right | Female | BC | 1928 | Pelt with manus attached |
| 5 | Left | Male | BC | 1959 | Pelt with manus attached |
| 6 | Right | Male | BC | 1947 | Pelt with manus attached |
| 7 | Right | Male | BC | 1989 | Pelt with manus attached |
| 8 | Left | No data | BC | No data | Pelt with manus attached |
| 9 | Right | Female | No data | 2009 | Manus Only |
| 10 | Right | Female | No data | 2000 | Manus Only |
| 11 | Right | No data | No data | No data | Manus Only |
| 12 | Right | Female | BC | 1984 | Manus Only |
| 13 | Right | Male | BC | 2013 | Manus Only |
| 14 | Right | Male | BC | 2016 | Manus Only |
| 15 | Right | Male | No data | 1998 | Manus Only |

**FIGURE S3** Actual badger manus and the 3D scan models. The numbers to the left of the figures correspond to the badger specimen numbers

## Morphological characteristics of badger manus and claws

Morphological characteristics of the badger manus and claw were obtained and are presented in the following section. Each of the five digits on the manus was given a number (Figure 3a). The manus width (*w_m_*) was measured from the outer side of digit I to the outer side of digit V (Figure S4a), and thickness (*t_m_*) was measured along Section A-A as the linear distance at the centre of the middle digital pad from the ventral to the dorsal aspect of the manus (Figure S4b). For the claws, the length (*l_c_*) was the linear distance from the proximal to the distal end of the claw, and the width (*w_c_*) and the thickness (*t_c_*) were both measured near the proximal end along Section B-B (Figure S4c). The claw of digit III is the most functional claw in digging (Teixeira-Filho et al., 2001; Tulli et al., 2009; Vrcibradic & Rocha, 1996). For the digit III claws, additional characteristic, its radius of curvature (*R*) was obtained by the following steps (Figure S4d): (1) the midpoint of the claw base was identified; (2) a line perpendicular to the claw base and passing through its midpoint was drawn; (3) a circular arc tangent to the line in step 2 was drawn from the midpoint of the claw base to the claw tip; and (4) the equivalent *R* of the circular arc was considered to be the representative *R* for the claw.

| **(a)** | **(b)** | **(c)** |
| --- | --- | --- |
|  |  |    |
| **(d)** | | |
|  | | |

**FIGURE S4** Badger manus and morphological characteristics. (a) Ventral aspect of the manus model showing the manus width (*w_m_*). (b) Lateral aspect of section A-A showing the manus thickness (*t_m_*). (c) Definitions of the claw length (*l_c_*), thickness (*t_c_*), and width (*w_c_*). (d) Measurements of the radius of curvature (*R*)

The characteristics of manus and claws were compared between the male and female badgers. Overall, the average manus width and thickness were 39 and 20 mm, respectively. The differences in manus width (*w_m_*) and thickness (*t_m_*) were not significant (Figure S5a) and neither were the differences in claw length (*l_c_*), width (*w_c_*), and thickness (*t_c_*) (Figure S5b). On average, the claw of digit III of male badgers had a radius of curvature (*R*) of 27.1 mm which was significantly larger than that of females (*R*=20.4 mm). Digits II, III, and IV were longer, wider, and thicker among the five claws (Figure S5c). Digit III was the longest (*l_c_*=24.1 ±3.2 mm), widest (*w_c_*=8.6 ±0.8 mm), and thickest (*t_c_*=4.3 ±0.5 mm). This digit is assumed to penetrate the substrate first and exit last during digging (Birn-Jeffery et al., 2012), while digits I and V are sub-functional, as observed in other mammals (Lull, 1904). Long claws are found to be essential for digging (Stein, 2000), and badger claws are observed to be relatively longer among burrowing animals.

The cross-sectional robustness of the badger claw provides several advantages for digging, including stabilizing the joint and preventing claw dislocation to strengthen the claw and withstand claw-soil interaction forces (Hopkins & Davis, 2009). From a mechanical perspective, the claw base is subjected to the maximum bending load and the largest cross-sectional area (reflected by *w_c_* and *t_c_*) of the claw at the base helps reduce the internal bending stresses. Also, the elliptical cross-sectional shape (*w_c_* exceeding *t_c_*) when compared to square or circular shapes, has a larger mechanical advantage against bending based on solid mechanics theories. Thus, from a biomechanical perspective, the cross-section shape of the claw is a fundamental adaptation for high levels of strength for soil cutting.

| **(a)** | **(b)** |
| --- | --- |
|  |  |
| **(c)** | |
|  | |

**FIGURE S5** Measured morphological characteristics. (a) Comparisons in width (*w_m_*) and thickness (*t_m_*) of manus between male and female badgers. (b) Comparisons in length (*l_c_*), width (*w_c_*), thickness (*t_c_*), and radius of curvature of claw (*R*) between male and female badgers. (c) the length (*l_c_*), width (*w_c_*) and thickness (*t_c_*) of the claw of different digits. Means that correspond to different letters are significantly different within each measured variable at P<0.05 according to Duncan’s Multiple Range tests; the error bars represent the standard deviations

## Creation of artificial claws and manus for simulations

First, the most representative manus (Specimen No. 12) was selected. The dimensions of this specimen had the least overall relative error (4.5%) when compared to the average values of the morphological characteristics of all 15 specimens. Then, based on this specimen, twelve different radii of curvature (*R*) values are generated, and they are 10, 15, 20, 25, 30, 35, 40, 45, 50, 55, 60, and 65 mm, while keeping the arc length the same as the representative claw (Figure 3b). This ensured that *R* was the only varying factor examined. Correspondingly, twelve artificial claws were created in *Geomagic Studio* (Figure 3c).

Based on the same specimen, 1-, 2-, 3-, 4-, 6-, 7-, and 8-digit artificial manus were created using *Geomagic Studio* (Figure 5b). The manus reconstruction took the following four considerations: (1) all manus included digit III because the axis of the foot lies along this digit in most fossorial mammals, and digit III is assumed to have the highest functional significance for digging (Lull, 1904; Tulli et al., 2009); (2) digit I is the least important, and it tends to be the first to be removed (Lull, 1904); (3) it was assumed that digit V is the second most important; (4) for manus with an odd number of digits (7), digit III is in the middle; and for manus with an even number of digits (6 and 8); it was assumed that digit III is located closer to digit V. Digits are adducted and are close to each other before digging (Figure S6a), and digits are flexed to assist in soil digging (Figure S6b).

| **(a)** | **(b)** |
| --- | --- |
| **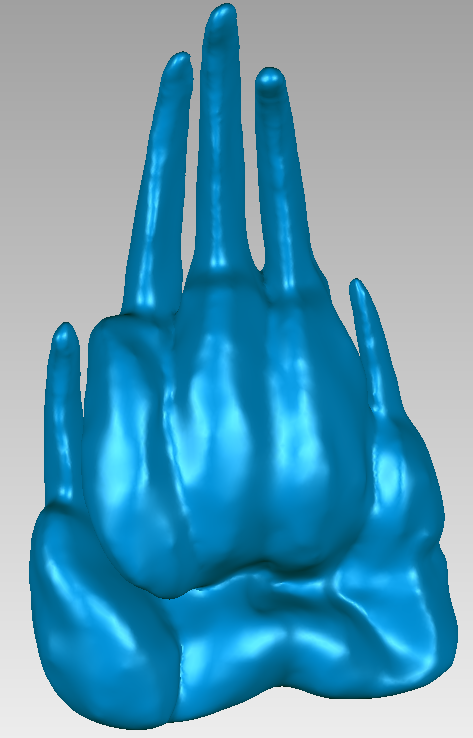** | **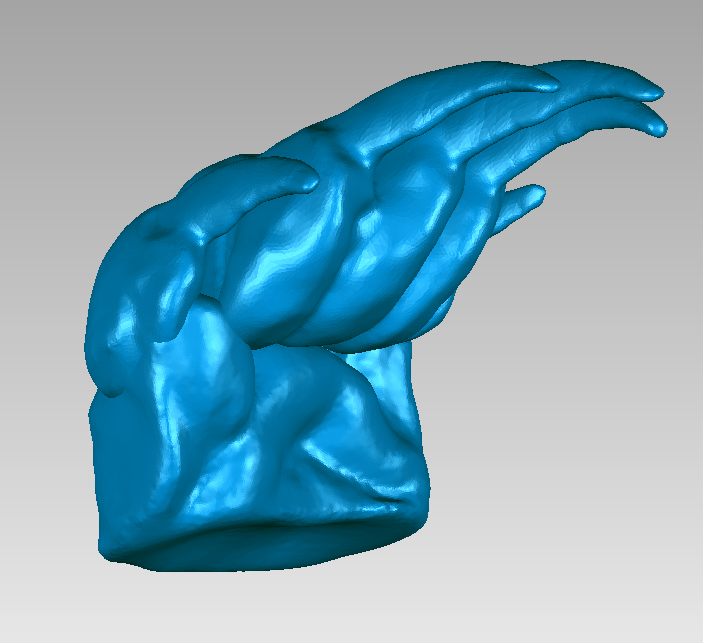** |

**FIGURE S6** Digit positions during digging in simulations (a) Adduction and (b) Flexion of the manus

## Generation of the motions of badger forelimb

Modelling manus-soil and claw-soil interactions require the input of the badger’s forelimb motions during digging. The motion was developed based on the data collected by Quaife (1978), who recorded the digging motions of live North American badgers. The data showed that the forelimb digging motion involved a power stroke (engaging with soil) and a retracting stroke (retracting the manus to the initial position). Only the motion of the power stroke was needed for the modelling in this study because no soil is displaced during the retracting stroke. The forelimb consists of brachium, ante-brachium, and metacarpus, and the corresponding lengths of these components were 10.2, 10.9, and 2.3 cm, respectively (Rose et al., 2014). The lengths of the digits and claws were obtained from image analyses of the 3D scans. Quaife (1978) provided information on the initial and final flexion/extension and rotational velocities of the forelimb components, and the total time to finish the power strokes for both soil cutting and digging. These details were used to track the position of each forelimb component at different times after the initiation of the power stroke and then generated plots from various aspects (lateral, dorsal, and frontal). Figure S7a shows the time elapsed at each instance after the initiation of the power stroke. The orientation of the X-Y-Z axis with respect to the body of the badger was defined in Figure S7b. The X-Y, X-Z, and Z-Y planes represent the lateral, dorsal, and frontal aspects, respectively. These aspects during soil cutting by a claw are illustrated in Figures S7c-e and those during soil digging by a manus are shown in Figures S7f-h. The strokes performed in soil digging have slower velocities than soil cutting, but they involve a wider range of arm motion to transfer the soil to the hind limbs effectively. Soil cutting involves faster strokes with less rotational displacement to effectively push the claws onto the soil surface. The positions of the forelimb components at different time points during digging and cutting are also reflected in the graphs (Figures S7c–h). Knowing the position and orientation of each forelimb component at different time points is essential to code and verify their movements in the soil cutting and digging simulation models.

| **(a)** | | | | **(b)** | | | |
| --- | --- | --- | --- | --- | --- | --- | --- |
|  | | | |  | | | |
| **(c)** | **(d)** | | | | **(e)** | | |
|  | | |  | | | |  |
| **(f)** | | **(g)** | | | | **(h)** | |
|  |  | | | |  | | |

**FIGURE S7** Motions of the badger forelimb developed based on the data collected by Quaife (1978). (a) Legend indicating which forelimb component the symbols represent and the time elapsed at each instance after the initiation of the power stroke; Dig-1/Cut-1 indicates the initial position, while Dig-5/Cut-5 corresponds to the final position of the forelimbs. (b) Orientation of the X-Y-Z axis with respect to the body of the badger (Quaife, 1978). (c–e) Forelimb motion performed by the claws during soil cutting from the lateral aspect [X-Y], dorsal aspect [X-Z], and frontal aspect [Z-Y], respectively. (f–h) Forelimb motion performed by the manus during soil digging from the lateral aspect [X-Y], dorsal aspect [X-Z], and frontal aspect [Z-Y], respectively

## Figures

| **(a)** | **(b)** | **(c)** | **(d)** |
| --- | --- | --- | --- |
|  |  | 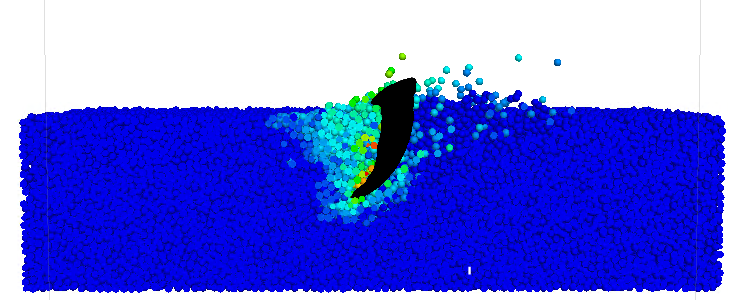 |  |
|  | 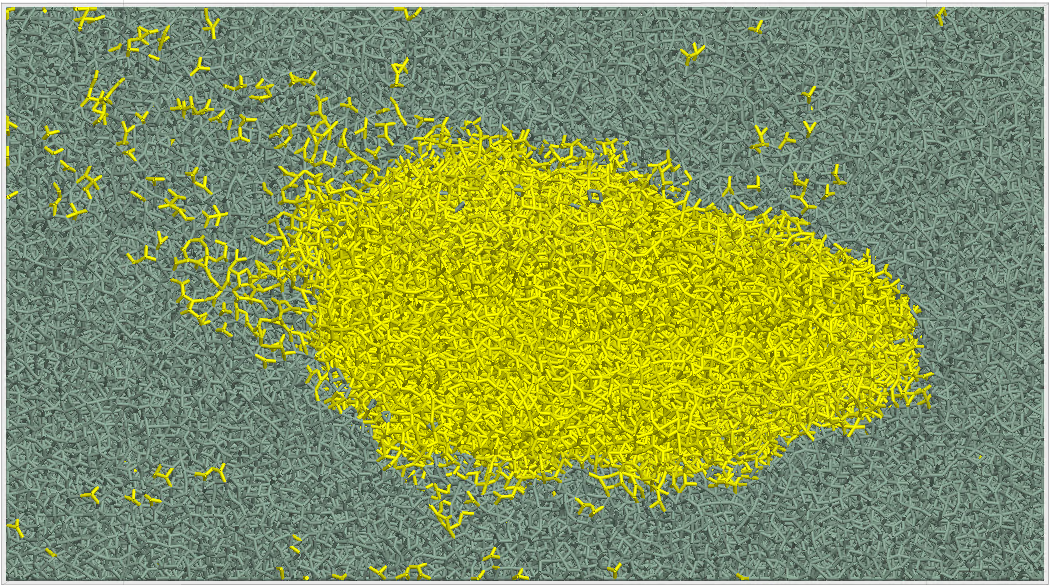 | 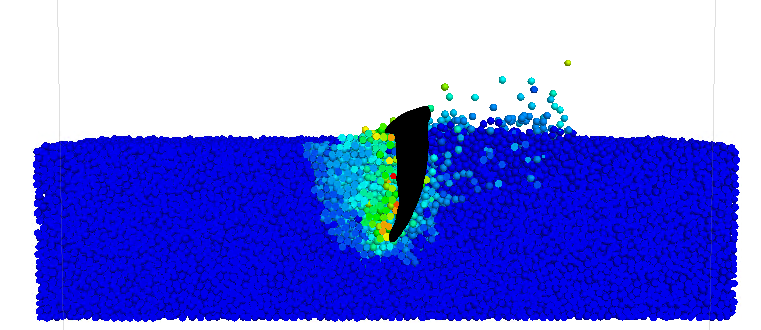 |  |
|  | 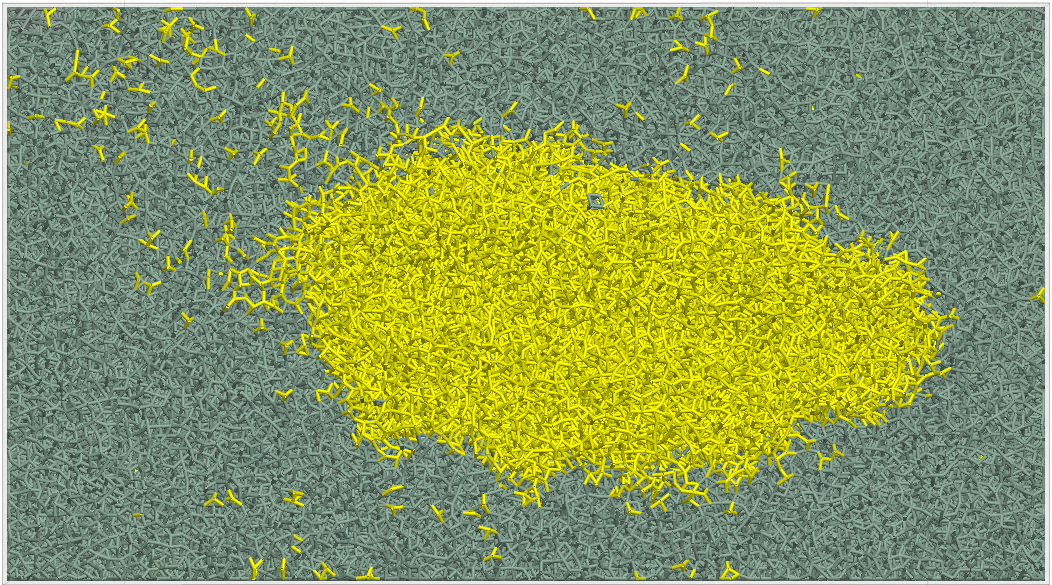 | 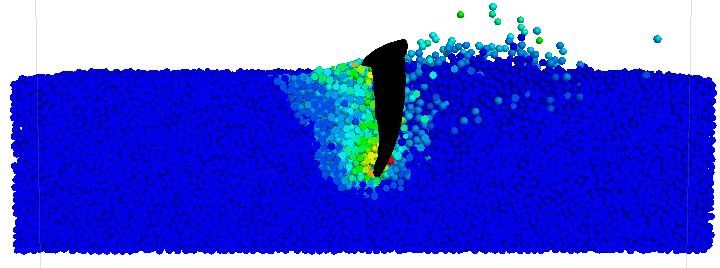 |  |
|  | 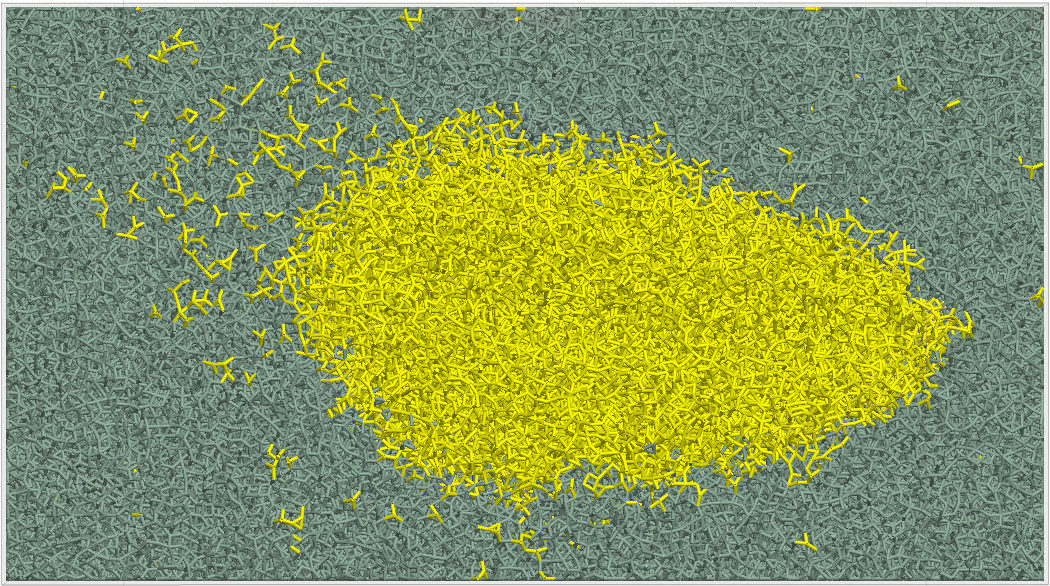 | 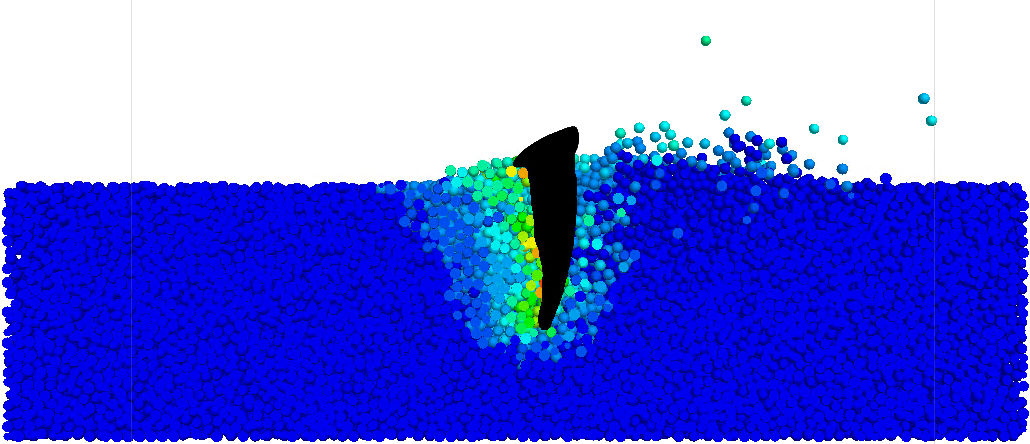 |  |
|  | 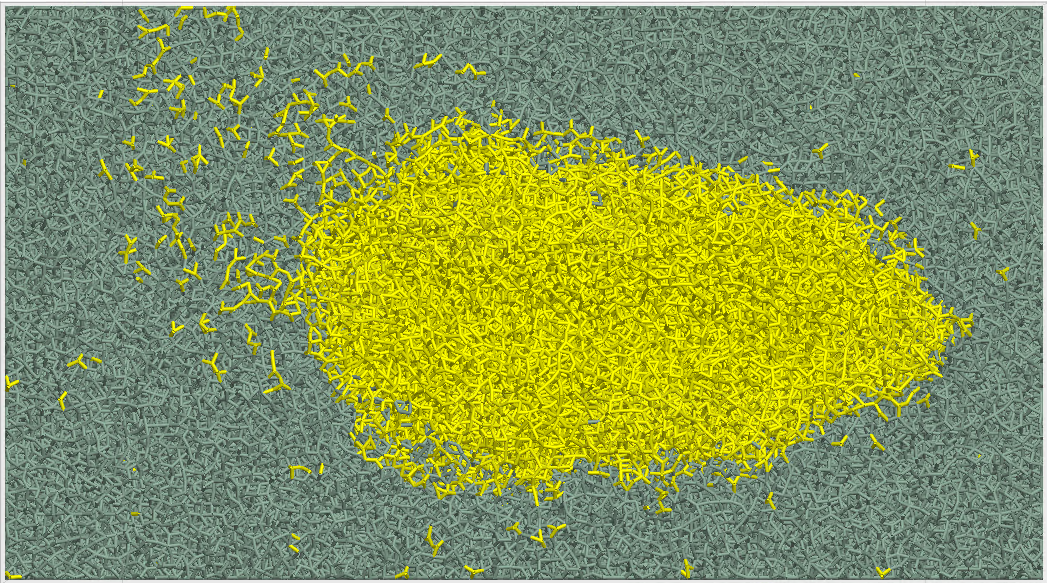 | 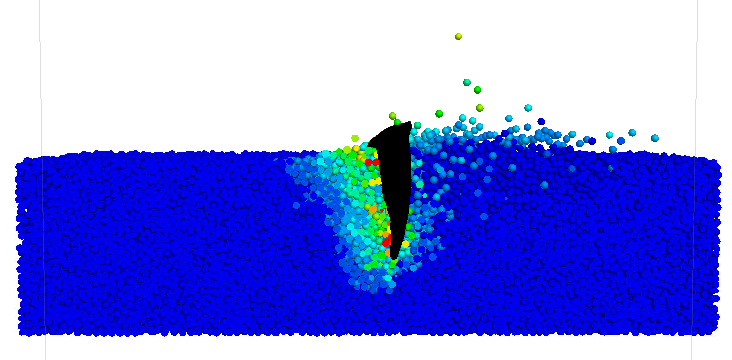 |  |

**FIGURE S8** Results of soil cutting performed by claws with different radii of curvature (*R*): 20, 30, 35, 40, and 45 mm. (a) Soil cutting force experienced by the claw. (b) Top view of the soil surface highlighting the cut soil. (c) Cross-sectional view of the soil particle velocity contours. (d) Legend showing the equivalent soil particle velocities of the colour contours

| **(a)** | **(b)** | **(c)** | **(d)** |
| --- | --- | --- | --- |
|  |  | 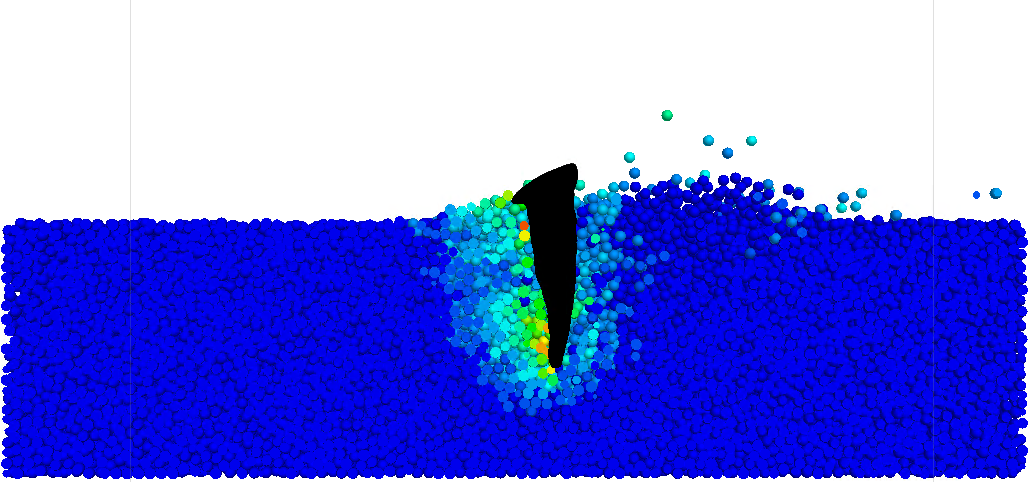 |  |
|  | 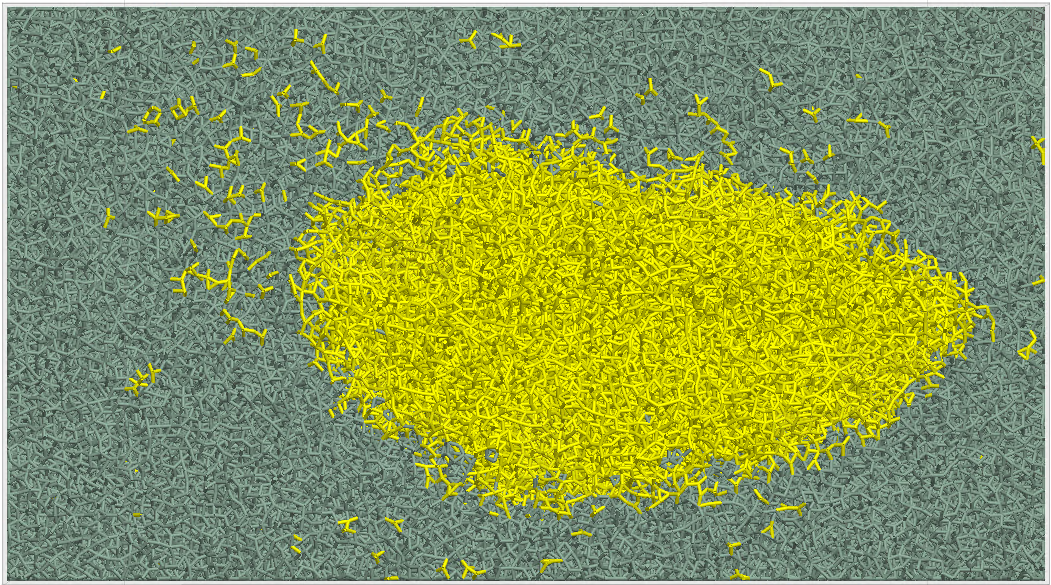 | 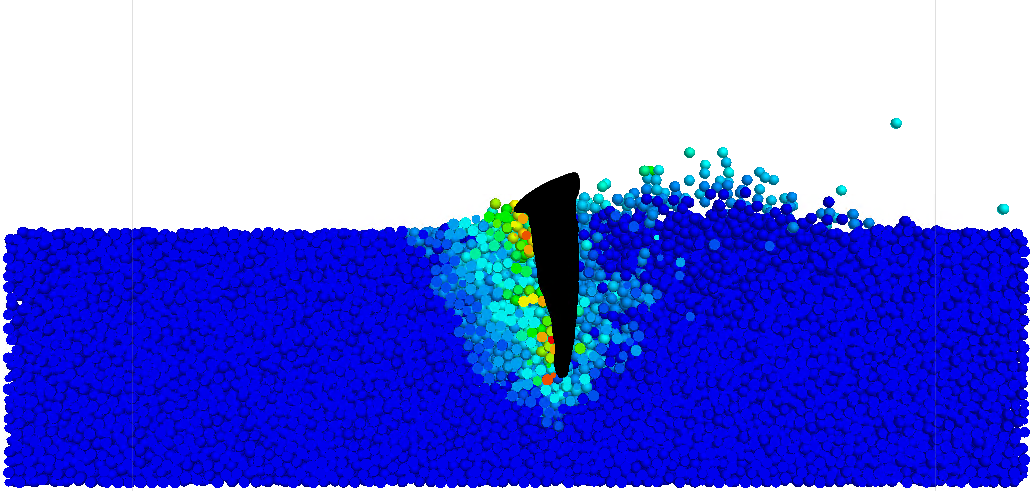 |  |
|  | 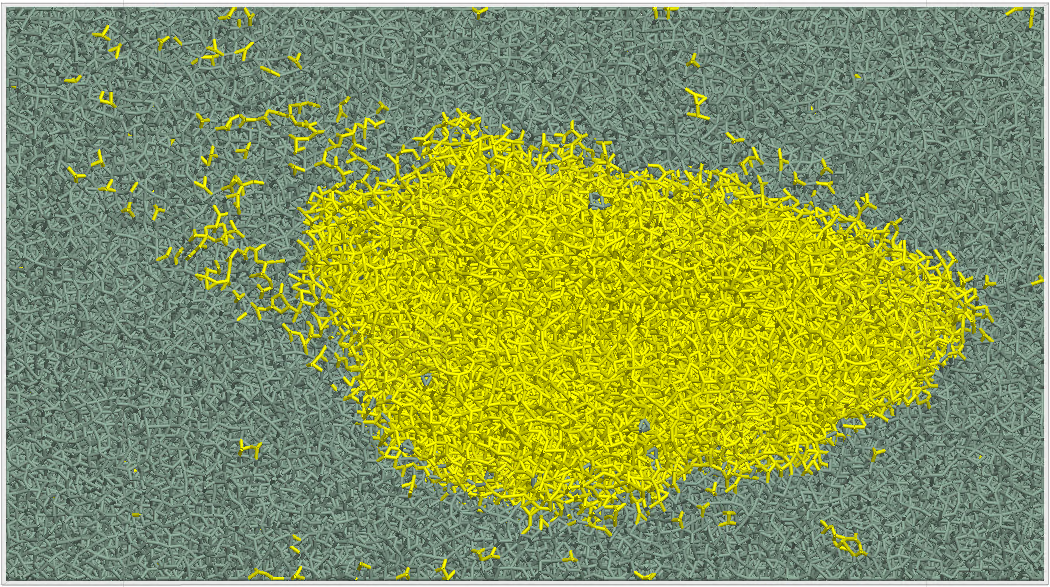 | 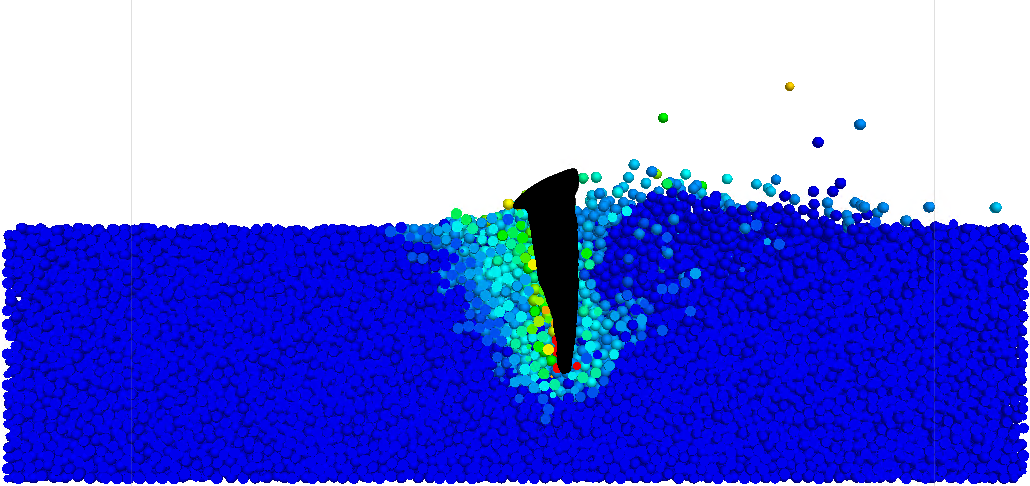 |  |
|  | 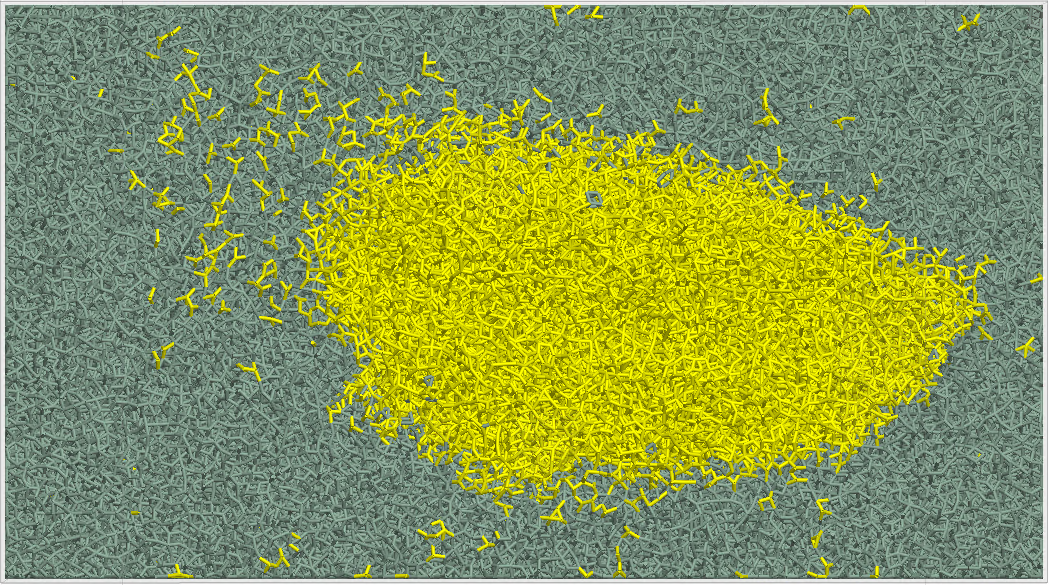 | 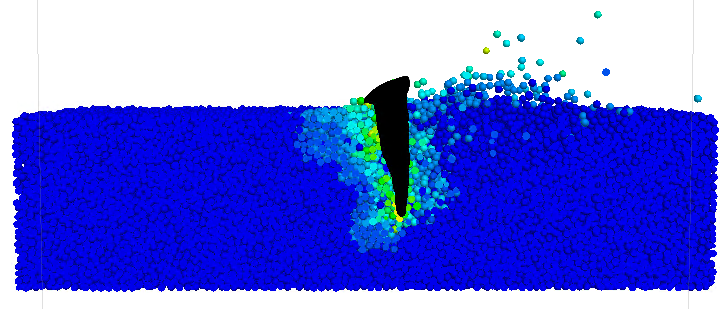 |  |

**FIGURE S9** Results of soil cutting performed by claws with different radii of curvature (*R*): 50, 55, 60, and 65 mm. (a) Soil cutting force experienced by the claw. (b) Top view of the soil surface highlighting the cut soil. (c) Cross-sectional view of the soil particle velocity contours. (d) Legend showing the equivalent soil particle velocities of the colour contours

| **(a)** | | **(b)** | **(c)** |
| --- | --- | --- | --- |
|  | 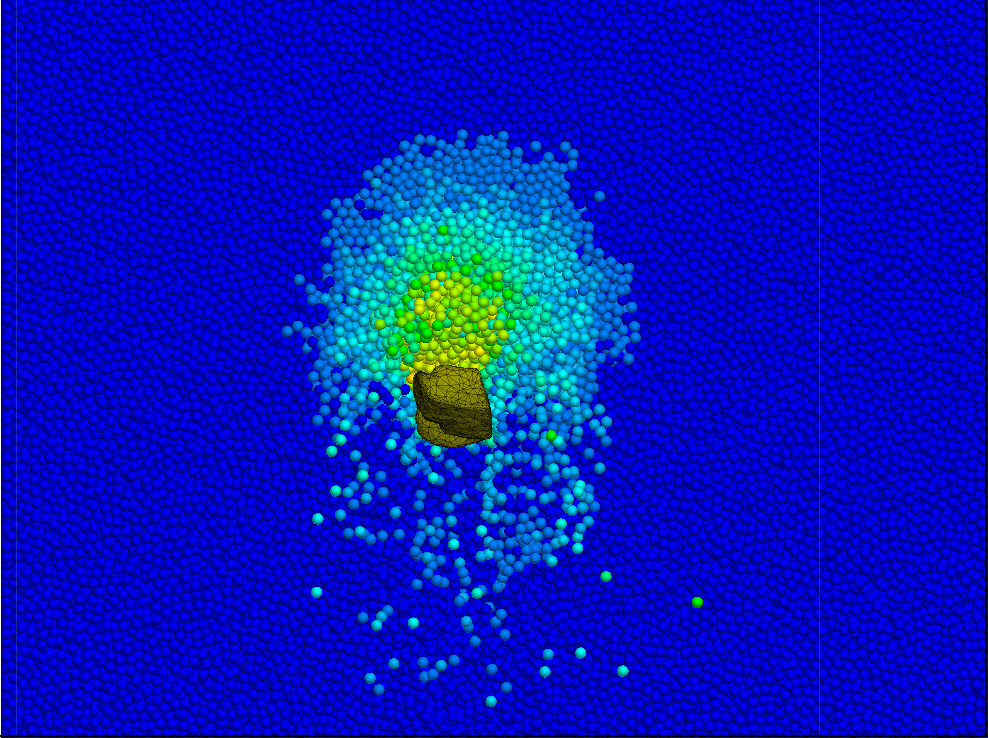 |  |  |
|  | 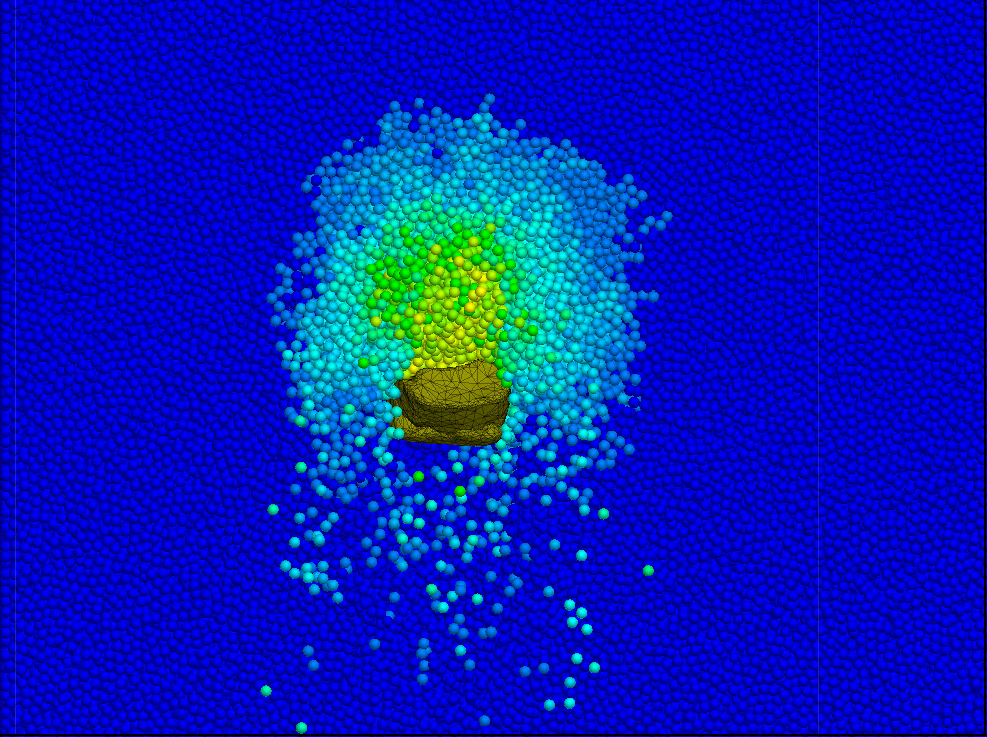 |  |  |
|  | 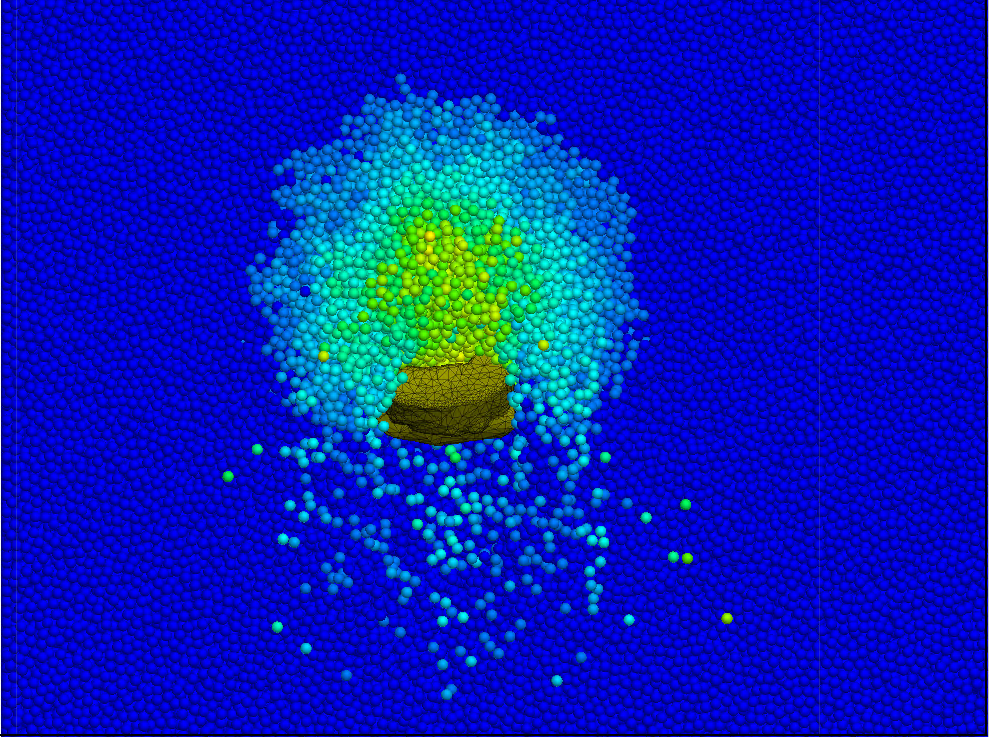 |  |  |
|  | 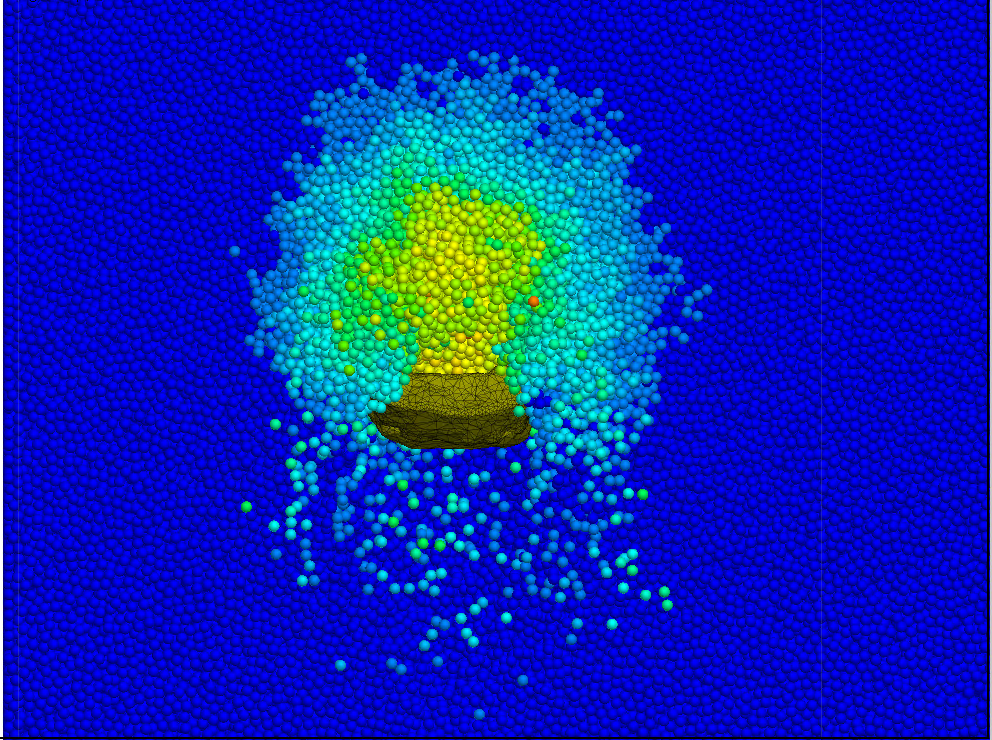 |  |  |
|  |  |  |  |

**FIGURE S10** Results of soil digging performed by manus with different numbers of digits: 2-, 4-, 6-, and 7-digit manus. (a) Screenshots of the soil displaced showing the velocity contours of the particles from the cross-sectional and top views. (b) Legend showing the equivalent soil particle velocities of the colour contours in Figure S5a. (c) Accumulated mass of displaced soil after four strokes of the manus digging motion. (d) Resistance force exerted on the manus during four strokes of the manus digging motion

## References

Apps, C. D., Newhouse, N. J., & Kinley, T. A. (2002). Habitat associations of American badgers in southeastern British Columbia. *Canadian Journal of Zoology*, *80*(7), 1228–1239. https://doi.org/https://doi.org/10.1139/z02-119

Birn-Jeffery, A. V., Miller, C. E., Naish, D., Rayfield, E. J., & Hone, D. W. E. (2012). Pedal claw curvature in birds, lizards and mesozoic dinosaurs - complicated categories and compensating for mass-specific and phylogenetic control. *PLoS ONE*, *7*(12). https://doi.org/https://doi.org/10.1371/journal.pone.0050555

Hopkins, S. S. B., & Davis, E. B. (2009). Quantitative morphological proxies for fossoriality in small mammals. *Journal of Mammalogy*, *90*(6), 1449–1460. https://doi.org/https://doi.org/10.1644/08-MAMM-A-262R1.1

Lull, R. S. (1904). Adaptations to aquatic, arboreal, fossorial and cursorial habits in mammals. *The American Naturalist*, *38*(445), 1–11. https://doi.org/https://www.jstor.org/stable/2454914

Mahadi, M. R., Chen, Y., & Botha, P. (2017). Instrumented Soil Bin for Testing Soil-Engaging Tools. *Applied Engineering in Agriculture*, *33*(3), 357–366. https://doi.org/http://elibrary.asabe.org/abstract.asp?AID=47756&t=3&dabs=Y&redir=&redirType=

McKyes, E. (1985). *Soil Cutting and Tillage*. Elsevier.

Quaife, R. (1978). *The Form and Function of the North American Badger in Relation to its Fossorial Way of Life*. University of Calgary.

Quinn, J. H. (2008). *The ecologiy of the American badger Taxedia taxus in California: assessing conservation needs on multiple scales*. University of California, Davis.

Rose, J., Moore, A., Russell, A., & Butcher, M. (2014). Functional osteology of the forelimb digging apparatus of badgers. *Journal of Mammalogy*, *95*(3), 543–558. https://doi.org/https://doi.org/10.1644/13-MAMM-A-174

Sadek, M. A., & Chen, Y. (2015). Feasibility of Using PFC3D to Simulate Soil Flow Resulting from a Simple Soil-Engaging Tool. *Transactions of the ASABE*, *58*(4), 987–996. https://doi.org/http://elibrary.asabe.org/abstract.asp?aid=46361&t=3&dabs=Y&redir=&redirType=

Stein, B. R. (2000). Morphology of Subterranean rodents. In E. Lacey, J. Patton, & G. Cameron (Eds.), *Life Underground: The Biology of Subterranean Rodents* (pp. 19–60). The University of Chicago Press.

Teixeira-Filho, P., Rocha-Barbosa, O., Paes, V., Carvalho Ribas, S., & de Almeida, J. R. (2001). Ecomorphological relationships in six lizard species of Restinga Da Barra De Maricá, Rio De Janeiro, Brazil. *Revista Chilena de Anatomía*, *19*(1). https://doi.org/http://dx.doi.org/10.4067/S0716-98682001000100007

Tulli, M. J., Cruz, F. B., Herrel, A., Vanhooydonck, B., & Abdala, V. (2009). The interplay between claw morphology and microhabitat use in neotropical iguanian lizards. *Zoology*, *112*(5), 379–392. https://doi.org/http://dx.doi.org/10.1016/j.zool.2009.02.001

Vrcibradic, D., & Rocha, C. F. D. (1996). Ecological Differences in Tropical Sympatric Skinks (Mabuya macrorhyncha and Mabuya agilis) in Southeastern Brazil. *Journal of Herpetology*, *30*(1), 60. https://doi.org/https://doi.org/10.2307/1564707
